# Low vision due to cerebral visual impairment: differentiating between acquired and genetic causes

**DOI:** 10.1186/1471-2415-14-59

**Published:** 2014-05-01

**Authors:** Daniëlle GM Bosch, F Nienke Boonstra, Michèl AAP Willemsen, Frans PM Cremers, Bert BA de Vries

**Affiliations:** 1 Bartiméus, Institute for the Visually Impaired, Zeist, The Netherlands; 2 Department of Human Genetics, Radboud University Medical Center, P.O. Box 9101, Nijmegen, HB 6500, The Netherlands; 3 Radboud Institute for Molecular Life Sciences, Radboud University Medical Center, Nijmegen, The Netherlands; 4 Donders Institute for Brain, Cognition and Behavior, Radboud University Medical Center, Nijmegen, The Netherlands; 5 Department of Pediatric Neurology, Radboud University Medical Center, Nijmegen, The Netherlands

**Keywords:** Acquired, Cerebral visual impairment, Genetic diseases, Visually impaired children

## Abstract

**Background:**

To gain more insight into genetic causes of cerebral visual impairment (CVI) in children and to compare ophthalmological findings between genetic and acquired forms of CVI.

**Methods:**

The clinical data of 309 individuals (mainly children) with CVI, and a visual acuity ≤0.3 were analyzed for etiology and ocular variables. A differentiation was made between acquired and genetic causes. However, in persons with West syndrome or hydrocephalus, it might be impossible to unravel whether CVI is caused by the seizure disorder or increased intracranial pressure or by the underlying disorder (that in itself can be acquired or genetic). In two subgroups, individuals with ‘purely’ acquired CVI and with ‘purely’ genetic CVI, the ocular variables (such as strabismus, pale optic disc and visual field defects) were compared.

**Results:**

It was possible to identify a putative cause for CVI in 60% (184/309) of the cohort. In the remaining 40% the etiology could not be determined. A ‘purely’ acquired cause was identified in 80 of the patients (26%). West syndrome and/or hydrocephalus was identified in 21 patients (7%), and in 17 patients (6%) both an acquired cause and West and/or hydrocephalus was present. In 66 patients (21%) a genetic diagnosis was obtained, of which 38 (12%) had other possible risk factor (acquired, preterm birth, West syndrome or hydrocephalus), making differentiation between acquired and genetic not possible. In the remaining 28 patients (9%) a ‘purely’ genetic cause was identified.

CVI was identified for the first time in several genetic syndromes, such as ATR-X, Mowat-Wilson, and Pitt Hopkins syndrome. In the subgroup with ‘purely’ acquired causes (N = 80) strabismus (88% versus 64%), pale optic discs (65% versus 27%) and visual field defects (72% versus 30%) could be observed more frequent than in the subgroup with ‘purely’ genetic disorders (N = 28).

**Conclusions:**

We conclude that CVI can be part of a genetic syndrome and that abnormal ocular findings are present more frequently in acquired forms of CVI.

## Background

Cerebral visual impairment (CVI) is one of the major causes of low vision in the developed world and accounts for 27% of the visually impaired children [1]. Visual impairment in CVI is due to a disorder in projection and/or interpretation of the visual input in the brain [2]. In the absence of absolute criteria for the diagnosis, the following definition is commonly used: CVI includes all visual dysfunctions caused by damage to, or malfunctioning of, the retrochiasmatic pathways in the absence of any major ocular disease [3]. A more practical definition includes an impairment of vision with normal function of the ocular structures and anterior visual pathways [4-6]. The main features of CVI during ophthalmological investigation are: impaired visual acuity, visual field defects, and abnormal visual behavior, while obvious ocular abnormalities are not found. This visual behavior consists of looking away from the target while reaching to it, looking past the target, staring into lights, and fluctuating visual performances. Fixation abnormalities can be observed, such as a prolonged time before fixating on a stimulus and intermittent fixation towards a stimulus [7,8]. The visual acuity can range from blindness to normal, and is in CVI due to perinatal causes correlated to the severity of the brain damage [9-11]. Crowding, the impairment of the ability to recognize objects in clutter, is present in more than 40% of the normally sighted CVI individuals, however, in individuals with CVI and low vision the percentage of crowding is unknown [10,12,13]. The occurrence of strabismus (37-73%) and nystagmus (12-73%) varies highly among previous reports [3,5,6]. In funduscopy a (segmental) pale optic disc can be seen (25-44%), whereas in premature born individuals a small optic disc or a more pronounced excavation can be found [14]. Remarkably, the latter could also be observed in individuals with mutations in *NR2F1*, which were recently identified [15]. Visual fields can be impaired, varying from partial visual field defects, to severe constriction of visual fields [16]. It has been demonstrated that visual evoked responses do not differ between individuals with and without CVI, although the responses can be abnormal [17]. In addition, fixation is often severely impaired in CVI and minimal fixation is necessary for visual evoked responses. Therefore, visual evoked responses cannot be used to diagnose CVI. Furthermore, difficulties with object or face recognition and visiospatial disorders can be observed [7,18]. Neuropsychological testing can be helpful to gain more insight in specific higher perceptual deficits in the individual, but are only applicable to children with a developmental age above two years and 9 months [19]. For a first screening of higher perceptual functions several questionnaires have been developed [10,20].

In general, the causes of CVI can be divided into acquired and genetic forms. The acquired forms can be derived prenatally (e.g. intrauterine infections), perinatally (e.g. ischemic brain injury), and postnatally (e.g. hypoglycemia or meningitis). Perinatal problems, often due to premature birth, are the most frequent causes of acquired CVI, occurring in up to two-thirds of the cases, whereas neonatal meningitis and/or encephalitis has been reported as important causes in the postnatal period (2.5-12%) [2,3,5,6,14]. Other acquired causes of CVI, such as head trauma and brain tumors are less common [3,4,6]. In complex cases, like children with severe epilepsy or hydrocephalus, it might be impossible to unravel whether CVI is caused by the seizure disorder or increased intracranial pressure or by the underlying disorder (that in itself can be acquired or genetic) [21,22]. CVI, either acquired or genetic, is often part of a more complex phenotype, and co-occurrence of non-ophthalmic impairments and disorders, such as intellectual disability or epilepsy, are common [3,6].

Knowledge about the occurrence of CVI in persons with or without intellectual disability can alter the way of approaching the person. For example, it is important to offer objects one by one or for a longer period of time, in case of crowding or fixation abnormalities. Moreover, rehabilitation of children with CVI can improve their overall function [23].

Although few studies reported on large series of 100 to 170 individuals with CVI, little attention is paid to whether genetic disorders are involved [3,5,6]. Several studies have been performed on higher perceptual deficits in more common genetic syndromes, such as Williams syndrome and Prader-Willi syndrome [24,25]. However, in the description of these patients in those reports a reduced visual acuity is not mentioned. Our goal was to get more insight into the genetic causes of CVI with low vision. Because in acquired forms of CVI the mechanism and the moment of disruption are different compared to CVI due to genetic causes, we also aimed to investigate whether different ocular phenotypes could be observed for genetic versus acquired forms.

## Methods

All individuals with CVI included in this study were seen in Bartiméus, an institute for diagnostics, rehabilitation and schooling of the visually impaired in the Netherlands, in the period 2002–2012. Assuming that the incidence of visual impairment in 3.1 million Dutch children aged 0–15 years is similar to the 8 ⁄ 100 000 per year reported in Scandinavia, approximately half of the annually recorded children with low vision in the Netherlands are seen at Bartiméus [1]. All children were referred to Bartiméus by medical specialists, i.e. paediatricians, rehabilitation physicians, ophthalmologists, general physicians and orthoptists. The children were referred for different reasons, mostly because of suspected visual impairment. A minority was referred for further diagnosis such as electrophysiology. The majority of children lived at home with their parents and were seen in the outpatient facilities of the Institute. All investigations were performed by a pediatric ophthalmologist with assistance of a technical ophthalmological assistant and/or orthoptist. Visual acuity was measured mono- and binocularly with correction of the refraction error under controlled light conditions. Monocular vision was measured by covering one eye by a special device for occlusion which could be added to the glasses worn by the patient. Visual acuity in young children or in individuals with a young developmental age was measured by forced preferential looking (Teller Acuity Cards, 55 cm), or with “LH Symbols” (3 m) [26]. The confrontational method with white Stycar balls, 5 cm, was used to estimate the visual fields. The balls on a stick were presented in all quadrants by a person behind the individual investigated. A person in front looked for response to the presented object: eye movements, pointing or a verbal response. Eye alignment, fixation, following, and visual behavior were observed. A handheld slit lamp was used to assess the anterior segment. In mydriasis, funduscopy and retinoscopy were performed, whenever the patient and parents agreed. But in case of lack of cooperation funduscopy and retinoscopy were not always possible. Electroretinography was rarely performed, because of developmental age or behavioral problems. CVI was diagnosed when there was no other ocular diagnosis which could explain the visual impairment or visual field defect, and/or typical features such as poor fixation or crowding, and/or CVI were found at neuropsychological investigation. Neuropsychological investigation of the visual functions was, however, not possible in a majority of the individuals, because of their developmental age. Although, there are tests available from 2^+9^ years onwards, the tests used in Bartiméus in the past 10 years were only applicable in patients with a developmental age above six years. Crowding was measured with the C-test at 5 m or with a LH Symbols version of the C-test on 40 cm as described in Huurneman et al. and was defined increased when the crowded ratio was ≥2 [13,27,28]. Additional inclusion criteria were a first visit over a 10 years period between 1-1-2002 and 1-1-2012 and low vision, defined as a visual acuity of ≤0.3 or <1.6 cycles/cm at 55 cm or a visual field radius of ≤30° degrees [1]. Under the age of three the visual acuity was measured by Teller Acuity Cards and defined as decreased, when the acuity in cycles/degree was below the normal range for their age reported by Courage and Adams [29]. Exclusion criterion was a second ocular diagnosis causing low vision, such as cataract or retinopathy of prematurity. Optic nerve atrophy was not an exclusion criteria, because it can occur as a result of retrograde transsynaptic degeneration in CVI [30]. Primary (hereditary) optic atrophy or congenital idiopathic nystagmus were excluded based on the history and results during ocular examination by the ophthalmologist. Bilateral amblyopia was excluded as refraction was measured and corrected if necessary.

The most recent ophthalmologic examination was used for further analyses, including binocular visual acuity, visual fields, strabismus, nystagmus, refraction error and the aspect of the optic disc. The use of vigabatrin was registered because of the risk of constriction of visual field [31]. To identify potential causes of CVI we evaluated the genetic investigations, risk factors during pregnancy, birth, and neonatal/childhood period, and reports on cerebral imaging.

For the comparison of the ophthalmological findings the data of the individuals with acquired causes and with a genetic diagnosis were used. To avoid confounding factors, which may contribute to CVI, additional criteria were used to select the subgroups for this comparison. Individuals with West syndrome and hydrocephalus as well as individuals with a genetic diagnosis in combination with a gestational age <37 weeks, unknown gestational age or acquired causes (e.g. perinatal problems) were excluded. For the statistical analysis the Mann–Whitney U test and the Fisher’s Exact test were used. A *p*-value below 0.05 was considered to be significant. To control for the false discovery rate at 0.05 we used the Benjamini-Hochberg method [32]. This study was approved by the Ethics Committee of the Radboud university medical center (Commissie Mensgebonden Onderzoek, regio Arnhem-Nijmegen). According to the Committee informed consent was not necessary, because all data were processed anonymously. Local approval of the institute was obtained. The study was conducted according to the tenets of the Declaration of Helsinki.

## Results

309 individuals with low vision fulfilled the criteria for CVI. The general information about the cohort is presented in Table 1 and in more detail in Additional file 1: Table S1. Almost half of the patients (151/309) had their first ophthalmological examination before the age of three years, whereas five individuals were over 18 years of age. 152 persons had more than one examination, and there was a median interval of 28 months between the first and the most recent examination. The persons without a second examination (n = 157) had a median age of 46 months (5 months - 45 years). In 20% (62/309) of the cohort there was a vision below 0.05 or 1.6 cycles/cm at 55 cm (Teller acuity cards) (Table 2). High refraction errors, myopia < -4 or hypermetropia > +4, were present in 25% (42/172) of the persons. In 77% (226/295) a strabismus and in 42% (113/270) a nystagmus was present. Visual field defects were found in 60% (149/249), consisting of constricted visual fields in half of the individuals. (Partial) pale optic discs were present in 44% (128/293). Fixation problems were present in 45% and fluctuating visual performances 31%.

**Table 1:** **General information**, **co**-**morbidities**, **etiology of the cohort**

**General information**	**Median (range)**
Age first examination (months)	36 (4 months- 45 years)
Age most recent examination (months)	53 (5 months – 45 years)
Birth weight (gram)	2980 (760–4750)
Gestational age (weeks)	39 (27–42)
	**Regarding N/Available N (%)**
Men	170/309 (55%)
Mortality	15/309 (5%)
Twins	20/309 (6%)
Gestational age <37 weeks	71/222 (32%)
Vigabatrin use	44/309 (14%)
Anomalies MRI-cerebrum	178/206 (86%)
**Co**-**morbidities**	**Affected N/Available N (%)**
Intellectual disability	298/309 (96%)
Motor impairment	288/294 (98%)
Hearing impairment	24/196 (12%)
**Etiology**	**Number (%)**
Perinatal problems*	70 (23%)
PVL*	35
Stroke*	32
Exogenic*	33 (11%)
Meningitis/encephalitis *	13
Congenital CMV infection*	6
Headtrauma*	6
Complication of operation/co-morbidity*	8
Genetic diagnosis*	66 (21%)
Hydrocephalus*	20 (6%)
West syndrome*	28 (9%)
Unknown	125 (40%)

*Can coexist.

**Table 2:** Findings of ophthalmological examinations

**Ocular findings**	**Affected N/Available N (%)**
Myopia < -4	13/171 (8%)
Hypermetropia > +4	29/171 (17%)
Visual acuity < 0.05 or <1.6 cycles/cm at 55 cm	62/309 (20%)
Strabismus	226/295 (77%)
Nystagmus	113/270 (42%)
Eye movement disorders	100/283 (35%)
Visual field defect	149/249 (60%)
Hemianopsia	28/149 (19%)
Upper or lower visual field defect	37/149 (25%)
Constriction of visual field	84/149 (56%)
(Partial) pale optic disc	128/293 (44%)
**Visual behavior/higher visual disorders**	**Percentage**
Fluctuating visual performances	31%
Fixation abnormalities	45%
Looking away from the target	19%
Crowding	8%
Problems with object/face recognition	7%
Staring at lights	15%
Auditive stimuli dominates	9%

Co-morbidities were found in a high percentage, such as intellectual disability (96%, 298/309, IQ < 70 or developmental delay) and hearing impairment (12%, 24/196, hearing threshold above 25 dB) (ICD-10, http://www.who.int/classifications/icd/en/) (Table 1). In 32% (71/221) the gestational age was below 37 weeks, of which two-thirds were born before 35 weeks. In 67% of the cohort a MRI-scan was performed, which was reported normal in only 14% (28/206). Brain anomalies were either caused by a disturbance in the developmental process (e.g. lissencephaly, cortical dysplasia) and white matter disease (e.g. hypomyelinisation) or acquired (e.g. periventricular leucomalacia (PVL), stroke). We subdivided the patients in a normal MRI group and an abnormal MRI group and searched for the difference in ophthalmologic features. In the patients with an abnormal MRI scan more pale optic disc were seen (49% versus 14%, *p* = 0.001) (Additional file 2: Table S2).

It was possible to identify one or more possible causes for CVI in 60% of the cohort. In 23% there was evidence of severe perinatal problems, such as hemorrhage or infarctions, PVL or post-partum reanimation. In 11% exogenous factors led to brain damage and CVI, such as meningitis or encephalitis, a diabetic coma, or a complication during an operation. Hydrocephalus was present in 6% and West syndrome in 9% of the cohort.

A genetic diagnosis was reported in 21% (Table 3), of which 50% had a proven chromosomal aberration. In half of the persons with a genetic syndrome other factors, such as West syndrome or stroke, may have contributed to the CVI. In the other half the CVI might be associated with the genetic syndrome.

**Table 3:** Syndromic diagnosis in patients with CVI

**ID**	Diagnosis*	**GA**	**Contributing factors**	**Age at examination (months)**	**CVI previously reported**	**Evidence for genetic diagnosis**	**Gene**
269	Aicardi syndrome	36	W	50	[33]	C	
316	Aicardi syndrome	42	W	108		C	
173	Aromatic decarboxylase deficiency	32		38	[34]	C,M	
**319**	**ATR-X**	**38**		**70**		**C**,**G**	***ATRX***
55	CDG type 1a	41		22	[35,36]	C,M	
305	CDG type 1a	NA		25		C,M	
223	Citrullinaemia	NA		81		C,M	
**128**	**Coffin**-**Siris syndrome**	**41**		**84**		**C**	
**43**	**Cohen syndrome**	**38**		**53**		**C**	
433	Complex I deficiency	NA		15	[37,38]	C,M	
378**	Complex I deficiency	40		35		C,M	
403	Complex II deficiency	41		46	[37]	C,M	
5	Complex III deficiency	41	W	73		C,M	
266	Complex I and III deficiency	NA		50		C,M	
29	Complex II and III deficiency	42	S	48		C,M	
175	D2-hydroxyglutaaraciduria	NA		20	[39-41]	C,M	
425	Incontinentia Pigmenti	40	S, W	67	[42,43]	C	
184	Infantile neuroaxonal dystrophy	39		45	[44,45]	C	
325	Copper storage disorder	41		27	[46]	C,M	
263	Lissencephaly	NA		38	[47]	C,G	*DCX*
231	Marden-Walker syndrome	35		79		C	
**17**	**Mowat-Wilson syndrome**	**41**		**34**		**C**,**G**	***ZEB2***
215	Opitz C syndrome	NA		199		C	
216	Pelizaeus-Merzbacher syndrome	38		27	[4]	C,G	*PLP1*
**192**	**Pitt-Hopkins syndrome**	**40**		**35**		**C**,**G**	***TCF4***
85	Pontocerebellar hypoplasia type 2 Triple X	36		30	[48]	C, C,G	
238	Propion acidemia	36		117	[49]	C,M	
78	Rett syndrome	36		23		C,G	*MECP2*
99	Rett syndrome	38		20	[50]	C,G	*CDKL5*
172	Rett syndrome	NA		14		C,G	*CDKL5*
364	Rett syndrome	NA		197	[51,52]	C	
341	Tuberous sclerosis	NA	Compression from tubers	179	[4]	C	
41	Tuberous sclerosis	NA	Compression from tubers	165		C	
348	Vanishing white matter	NA		28	[53]	C	

*in an additional 33 individuals chromosomal aberrations were identified. **previously described in Morava et al. [54]. ATR-X syndrome = Alpha-Thalassemia X-linked intellectual disability syndrome, CDG = congenital disorder of glycosylation, C = clinical diagnosis, GA = gestational age, G = gene mutation, M = metabolic diagnosis, NA = not available, P = perinatal problems, S = stroke, W = West syndrome. The syndromes in the bold formatted rows are for the first time associated with CVI.

After excluding individuals who had multiple factors that could have contributed to CVI a subgroup (N = 108) remained, which allowed for the comparison of the ocular findings between individuals with a ‘purely’ acquired form of CVI (N = 80) and those with a ‘purely’ genetic form (N = 28). There were no significant differences between the groups according to the age at the ophthalmological examination (72 months versus 54 months, *p* = 0.333) or the use of vigabatrin (9% versus 7%, *p* = 1.00), of which a known side effect is visual field constriction defects (Table 4) [31]. The occurrence of blindness (16% versus 25%, *p* = 0.396), nystagmus (46% versus 35%, *p* = 0.357) or high refraction errors (myopia: 4% versus 0%, *p* = 0.567; hypermetropia; 6% versus 11%, *p* = 0.425) did not differ significantly between the groups. In the ‘purely’ acquired group, however, strabismus (88% versus 64%, *p* = 0.009), visual field defects (72% versus 30%, *p* = 0.001) and (partial) pale optic discs (65% versus 27%, *p* = 0.001) were observed more often than in the ‘purely’ genetic group. The type of visual field defects, concentric, hemianopsy, or upper or lower visual field defects, did not seem to differ between the two groups, but the numbers were too small to make definite conclusions.

**Table 4:** **Ocular findings in individuals with a** ‘**purely’ acquired or a** ‘**purely’ genetic cause**

	**Acquired (N = 80)**	**Genetic (N = 28)**	**Raw *p-*** **value**
Mean age most recent examination (months)	70 (SD 58)	54 (SD 39)	0.333
	**Affected N/Available N**	**Affected N/Available N**	
Men	48/80 (60%)	17/28 (61%)	1.00
Vigabatrin use	7/80 (9%)	2/28 (7%)	1.00
Abnormal MRI	55/56 (98%)	11/14 (79%)	0.023
Myopia < -4	3/80 (4%)	0/28 (0%)	0.567
Hypermetropia > +4	5/80 (6%)	3/28 (11%)	0.425
Visual acuity <0.05 or <1.6 cycles/cm at 55 cm	13/80 (16%)	7/28 (25%)	0.396
Strabismus	68/77 (88%)	18/28 (64%)	0.009*
Nystagmus	31/67 (46%)	9/26 (35%)	0.357
Visual field defect	47/65 (72%)	7/23 (30%)	0.001*
Hemianopsia	10/47	1/7	-
Upper or lower visual field defect	19/47	3/7	-
Constriction of visual field	18/47	3/7	-
(Partial) pale optic disc	51/78 (65%)	7/26 (27%)	0.001*

*p-Values represent differences that passed the Benjamini–Hochberg criterion (false discovery rate at 0.05).

## Discussion

We analyzed causes of CVI in a large cohort of 309 individuals with low vision in the Netherlands. Although CVI has also been described in individuals with a (sub-)normal visual acuity and higher perceptual deficits, within this study only patients were selected with a vision ≤0.3 or a visual field below 30 degrees [9,10,18]. For the purpose of this study, we excluded persons with major anterior ocular diseases, although CVI can also be present in those patients. In some persons only grating acuity (Teller Acuity Cards) could be measured. In general using grating acuity initially may lead to higher scores than the optotype acuity that can be applied later in development [55]. Although some children were investigated at a very young age, which might hamper the differentiation between CVI and delayed visual maturation (DVM), about half of the individuals had more than one ophthalmological examination, with a median interval of 28 months between the first and the most recent examination, whereas another quart of the persons were over 48 months allowing for an accurate clinical diagnosis. We hypothesized that in children with DVM there is a concordant delay in visual functions. In CVI, on the contrary, there may be discrepancies in the developmental stage of different visual functions. When this hypothesis is correct, it might be possible to differentiate earlier between CVI and DVM by signaling discrepancies in the profile of visual functions.

This is a retrospective study based on the medical files so that sometimes information is missing, for example on ERG, ophthalmological results or MRI scan. In addition, for some features (e.g. fixation) only abnormalities were reported, although this was probably assessed in all patients but not always recorded. However, we were able to collect a large group of patients and in the majority of these patients extended investigations were performed. The ocular findings in our cohort were comparable to previous reports, except for the visual field defects, of which a higher percentage was found in our study (60% versus 9%) [3,6]. In two-thirds of the patients an MRI - scan was performed. In only 14% of the individuals with an MRI scan of the brain, the images were reported to be normal (28/206), which is comparable with a previous study by Khetpal et al. [6]. The spectrum of MRI abnormalities in our cohort was broad: from structural abnormalities, such as corpus callosum dysplasia, holoprosencephaly or lissencephaly, to white matter abnormalities, such as hypomyelinisation and PVL. This shows that many different mechanism that lead to brain disease may cause CVI. In individuals with a normal MRI scan or only a thin corpus callosum, the pathogenesis was less clear. Pale optic discs were seen more frequently in the patients with reported anomalies on MRI scan. However, as the MRI scans were assessed by many radiologists with different expertise we are reluctant to draw firm conclusions.

In 60% of the individuals, we were able to identify the cause of the CVI, of which 32 persons were classified in more than one group. In 34% evidence of acquired problems, mainly perinatal, were found which is lower than the 42% - 67% previously reported [3,5,6]. This might be due to more stringent criteria used in our study, as only individuals with known brain damage on cerebral imaging, twin-twin transfusion syndrome or postpartum resuscitation were classified as perinatal problems.

In 40% of the patients we could not identify a cause for CVI. This can be due to missing information in the medical file, a not recognized acquired cause (for example an unrecognized CMV infection during pregnancy), a not identified genetic cause or a combination of several minor acquired and genetic risk factors.

In 21% of the individuals a genetic diagnosis was obtained, of which 50% had proven chromosomal aberrations (Table 3). As the techniques to identify underlying genetic causes are improving rapidly, this 21% is probably an underestimation.

We observed various genetic syndromes for which an association with CVI has previously been reported: trisomy 21, congenital disorder of glycosylation (CDG) type 1A, complex II deficiency, copper storage diseases, infantile neuroaxonal dystrophy, Pelizaeus-Merzbacher syndrome and Rett syndrome [4,35-37,44-46,50-52,56-58]. However, for the following genetic syndromes such association was not reported so far: Alpha-Thalassemia X-linked intellectual disability syndrome, Coffin-Siris syndrome, Pitt-Hopkins syndrome, Mowat-Wilson syndrome and Cohen syndrome.

In some of these syndromes retinal diseases have been described, for example in Cohen syndrome or CDG type 1A [35,59]. In our cohort an electroretinography was often not available. However, as funduscopy did not reveal any retinal abnormalities, retinopathy was unlikely to cause visual impairment. In neurodegenerative diseases, such as complex II deficiency, optic atrophy can occur as a result of mitochondrial dysfunction, but these disorders affect the brain equally [37]. Moreover, retrograde transsynaptic atrophy may occur, making differentiation between CVI and visual impairment due to optic atrophy in these disorders difficult.

Although CVI might still be a coincidental finding in the above mentioned genetic syndromes, most individuals with these or other syndromes have not systematically been tested for CVI. For example, visual acuity, visual behavior, and the findings at funduscopy are seldom reported in the clinical reports and children with an intellectual disability are not always seen by a pediatric ophthalmologist. Therefore, it is likely that CVI might be a consistent part of these syndromes, but a thorough assessment of the visual functions in more individuals with these syndromes is necessary to establish this association.

We were able to establish a diagnosis in the majority of CVI in our study population, consisting of genetic disorders and acquired causes. After exclusion of possible confounders a ‘purely’ genetic and a ‘purely’ acquired subgroup remained. Between those two different etiologies the ophthalmological examination revealed significant differences (Table 4). Strabismus, visual field defects and (partial) pale optic disc were significantly less common in the genetic group compared to the acquired group. This might be explained by the localization of the defect as in the acquired group, the most important cerebral damage was PVL and stroke, both mainly localized in the optic radiations. Damage to the optic radiations can cause visual field defects and retrograde optic disc pallor [14,30,60]. In the genetic group the optic radiations are probably less severely affected and a more overall dysfunction of the brain and/or cortical anomalies might have resulted in the visual impairment. This theory is supported by the observation that normal optic discs are seen more often in individuals with cortical lesions compared to subcortical lesions [60]. A comparable mechanism might result in the higher frequency of strabismus in the acquired group. To guide the alignment of the visual axes the binocular connections in the striate cortex play an important role [61]. When the input to the striate cortex is disturbed by prism-glasses, strabismus develops in primates [62]. So it can be expected that injury to the optic radiations by stroke or PVL, which also disrupts the input to the striate cortex, leads to strabismus. Although there are more mechanisms that cause strabismus, the above mentioned probably accounts for the difference between the two groups.

## Conclusions

In conclusion, we presented a large cohort of mainly children with CVI and impaired vision. Genetic disorders were present in 21% of patients and an association between the disorder and CVI is considered. CVI patients with genetic disorders showed an overall different and milder ocular phenotype: less frequently strabismus, visual field defects and pale optic discs. Moreover, we observed CVI for the first time in several known genetic syndromes making the screening of visual functions in those syndromes recommendable, to ensure the best approach and clinical care to the often multiple disabled persons. Also clinical genetic investigation should be considered in persons with CVI without evidence of an acquired cause. Awareness of CVI as part of a genetic disorder is currently increasing, and the techniques to identify the underlying genetic defect are improving rapidly. Therefore, it is likely that more genetic defects will be identified to be associated with CVI in the near future.

## Abbreviations

CVI: Cerebral visual impairment; DVM: Delayed visual maturation; PVL: Periventricular leucomalacia.

## Competing interests

The authors declare that they have no competing interests.

## Authors’ contributions

DGMB performed the acquisition of the data, conducted the data analysis and wrote the manuscript. FNB and FPMC contributed to the planning of the study. FNB, FPMC, BBAdV and MAAPW contributed to the analyses of the data and the critical revising of the manuscript. All authors read and approved the final manuscript.

## Pre-publication history

The pre-publication history for this paper can be accessed here:

http://www.biomedcentral.com/1471-2415/14/59/prepub

## Supplementary Material

Additional file 1: Table S1 Phenotype information of all patients involved. Click here for file

Additional file 2: Table S2 Ocular findings in individuals with a normal or abnormal MRI scan of the brain. Click here for file
